# Medical History from the Earliest Times

**Published:** 1893-03-18

**Authors:** E. T. Withington


### Medical History from the Earliest Times.

By E. T. Withington, M.A., M.B. Oxon.

### XLVII.—PARACELSUS.

The most striking figure among the physicians of the sixteenth century, perhaps in the whole of medical history, is Theophrastus von Hohenheim, called Paracelsus (1493-1541), to whom his enemies in his lifetime, and his admirers of a later day, have given the title "Lutherus Medicorum." Biographical details may readily be found elsewhere, and space will only suffice for a brief consideration of the importance of Paracelsus in medical history, and of the system which he attempted to substitute for that of Galen.

He tells us that, after travelling through nearly all Europe, and investigating the art of healing as practised not only by physicians but also by surgeons, women, conjurors, alchemists, monks, and in the houses of rich and poor, he came to the conclusion that medicine is uncertain, varying, and worthless—in short, a delusion of evil spirits. He had determined to give up the study altogether when his eye fell upon the words of the Gospel—"They that are whole need not a physician, but they that are sick"—and his estimate of medicine was at once completely reversed. An art sanctioned by Christ Himself could have nothing to do with evil spirits, but must, like all things which come from God, be certain, perfect, and perpetual. Its practitioners, however, had gone hopelessly astray, and it was reserved for him (Paracelsus) to show them the right path. This he proceeded to do with an extravagance of conceit and virulence of abuse which soon produced their natural results — hatred and persecution.

The only ancient physicians he respects are Apollo (!) Machaon (!) and Hippocrates; as for Galen, "He cannot boast of a single experiment, but learnt everything from others. He opposes nature in all, and is therefore a liar who can do nothing but collect pearls and turn them into pebbles; wherefore he is in the lowest hell, whither his disciples will follow him." His own colleagues he declares are whited sepulchres, and there is no hope for the elder ones, who are like untrained old dogs, and can learn nothing; but for the benefit of the younger he will expound the true and divine medicine, which rests upon four "pillars" so firmly based that heaven and earth shall pass away before it perishes or disappears.

The first of these pillars is Philosophy, which Paracelsus rates as highly as any ancient dogmatist. "Philosophy is the gate of medicine, and they who go not in thereat climb in by the roof and become thieves and murderers." This philosophy, however, is not that of Aristotle, which he compares to the froth and scum on the pot, containing perhaps some flavour of the broth, but fit only for dogs and cats; it comprises the whole circle of the sciences, physical and magical, especially the latter. An important division of it is "anatomy," not the dissection of bodies, which is a "boorish anatomy," practised by "Italian jugglers," and giving rise to nothing but error; but the "anatomy of the essence," an analysis, imaginary rather than chemical, of the intimate composition of man into its mystical ingredients "salt," "sulphur," and "mercury," and the recognition of the "forms" of disease, with the corresponding "forms" of their remedies. Like the "philosophy" generally, this knowledge is acquired not so much by the intellect as by the illumination of the Holy Spirit.

The second pillar of medicine is Astronomy. "No one can be a good physician who is not skilled in astronomy." The stars, indeed, have no influence on human temperament or fate; Nero would have been a savage and Helen an improper person, though there had been no such planets as Mars and Venus; in these respects a child's mother is its planet and star. But they do cause diseases by their exhalations, as for instance the sun by excessive heat, and it is no use trying to cure an astral disease while its star is in the ascendant. Therefore let physicians cease poking their noses into excrement, and lift up their eyes to the heavens, where they will find the fundamental principles of their art, and the path to true therapeutics.

The third pillar is Alchemy. "Without a perfect knowledge of alchemy the physician will use all the resources of his art in vain." Paracelsus classes here all attempts to improve natural substances. Thus the baker and weaver are alchemists; but its great use in medicine is to separate the quintessences of drugs, a foreshadowing of the modern search for "active principles." An alchemist, or "archeus," resides in the human stomach and presides over digestion, separating the poisonous from the nutritious part of the food, and when a human alchemist has attained similar skill he will be perfect in his art.

The fourth pillar is the Virtue of the physician; for the virtuous only are permitted to penetrate into the innermost nature of man and the universe, a power possessed in great perfection by Paracelsus, but found in very few besides, even among his own pupils, only ten in some hundreds of whom, he says, were at all satisfactory.

Man, according to Paracelsus, is a microcosm—that is, he contains elements representing every part of the universe. "There is nothing in heaven and earth which is not in man, and God, who is in heaven, is also in man." Diseases are caused or cured by the action of the various constituents of the universe (the macrocosm) on their corresponding parts in man (the microcosm), from which it follows that the physician must take all knowledge as his province, and that medicine is the highest and widest of sciences since it includes them all from theology downwards. Elsewhere he distinguishes five "Beings" (Entia) which are the causes of all diseases, and each of which can produce any disease, there being thus five kinds of jaundice, five kinds of dropsy, and so on. "When a physician, therefore, finds himself in the presence of a paralytic, he must, before all things, discover which 'Ens' has produced the paralysis." The first four are the "Ens Astrorum," or influence of the stars; the "Ens Veneni," or poison; the "Ens Naturale," or disturbances arising in the body itself; and the "Ens Spirituale," or spiritual agencies. But after carefully describing and distinguishing these, Paracelsus tells us that all this applies only to Turks, Saracens, and other infidels; for Christians and Jews there is but one cause of sickness, the "Ens Dei," or direct action of God, who sends diseases as purgatorial punishments. When the predestined hour of release comes, He either cures the patient directly, or through a physician, most of whom, however, resemble the demons of purgatory, and are sent to increase the torments of the sick.

